# Characterizing the hum of hovering animals

**DOI:** 10.7554/eLife.68072

**Published:** 2021-04-19

**Authors:** Robert Niese

**Affiliations:** College of Arts and Sciences, Pacific UniversityForest GroveUnited States

**Keywords:** bioacoustics, calypte anna, flapping wing, flying animals, insects, birds, bats, Other

## Abstract

The sounds of flying animals, such as the hum of a hummingbird as it hovers, are influenced by the unique forces generated by the flapping of their wings.

**Related research article** Hightower BJ, Wijnings PWA, Scholte R, Ingersoll R, Chin DD, Nguyen J, Shorr D, Lentink D. 2021. How oscillating aerodynamic forces explain the timbre of the hummingbird's hum and other animals in flapping flight. *eLife* **10**:e63107. doi: 10.7554/eLife.63107

Flight is an inherently noisy form of locomotion. For example, the sounds generated by a flock of pigeons can tell a listener exactly how urgently they are flying and their precise position (Clark, 2016; Larsson, 2012). Some species have even evolved specialized feathers that produce extra sounds to communicate the difference between casual and emergency take-off events (Murray et al., 2017). The characteristic hum of a hummingbird and the buzz of a mosquito are both produced by the motion of their wings during flight. Yet it is poorly understood how the act of flapping can generate such distinct sounds.

During flight, flapping wings generate vertical (lift) and horizontal (drag) forces that oscillate with every wingbeat. These forces can be observed as changes in the air pressure around an animal as it flaps. Similarly, sound waves move through the air as small, rippling changes in air pressure. Using exciting new tools and techniques a team of reasearchers at Stanford University were able to directly measure both types of pressure fluctuations in live hummingbirds as they hovered. Now, in eLife, Ben J Hightower (Stanford University) and Patrick WA Wijnings (Eindhoven University of Technology), and their team led by David Lentink (Stanford University), report new insights into the link between oscillating lift and drag forces and the acoustic qualities of the hummingbird’s hum (Hightower et al., 2021).

By adapting a model for propeller noise (Lowson, 1965), the team were able to mathematically describe the relationship between a flapping wing and the sound pressure waves it produces. The model created by Hightower et al. combines information about a wing’s motion and the forces it creates to predict how a wing sound will radiate through the air. These modeled sound waves are very similar to those observed in live hummingbirds and precisely matched some of their acoustic characteristics, like their pitch and loudness.

But as any musician can tell you, there is more to sound than just its pitch and loudness. The timbre, or acoustic quality, of a sound is the primary reason why different instruments playing the same note sound distinct from one another (Figure 1A and B). The simplest physical attribute of a sound that can help define its timbre is its harmonic content – that is, the distinct tones that are naturally produced beyond the original tone, or fundamental frequency, as it is played. The number and loudness of these harmonic tones largely determines the timbre of a sound.

**Figure 1.**
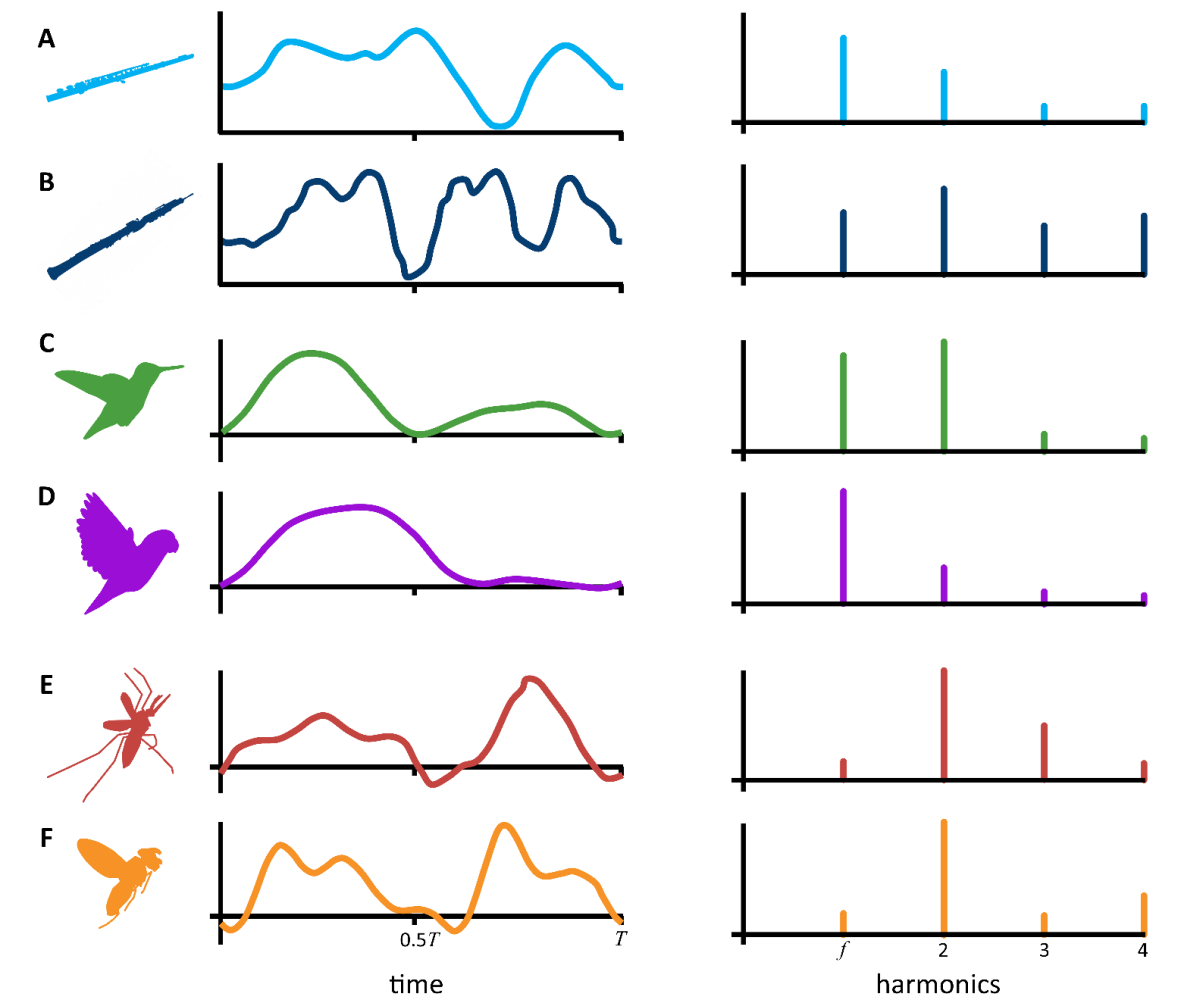
What determines the timbre of a sound? The cyclical motion of sound pressure waves or force pressure waves moving through the air can be represented by a waveform that repeats once every ‘T’ seconds (left side graphs). The number and relative height of the peaks and valleys of these waveforms directly influences the loudness of individual harmonics in the corresponding sound (right side graphs). Instruments like the flute (**A**) and oboe (**B**) playing the same note will produce sound pressure waves that repeat at the same rate (T) but have differing waveforms. This makes the harmonic content, the timbre, of the notes different. Hightower et al. showed that hovering animals – such as hummingbirds (**C**), parrotlets (**D**), mosquitos (**E**), and compact flies (**F**) – also produce unique pressure waves which repeat each time they flap their wing. Here, the waveforms represent vertical, lift forces instead of sound, but the link between the pressure waves and their harmonic content is the same. For complex waveforms (**B, E, and F**) with many peaks and valleys, the corresponding harmonics tend to be dominated by harmonics that are louder than the fundamental frequency (*f*). Whereas, in simpler waveforms (**A, C, and D**), the fundamental frequency is usually the loudest harmonic.

Using their model, Hightower et al. were also able to determine the precise links between aerodynamic forces, wing motion, and the harmonic content of the hummingbird’s hum. They found that the loudness of the second and fourth harmonic tones corresponds to the generation of vertical forces which happens twice per wingbeat in hummingbirds – first on the downstroke and then again on the upstroke (Warrick et al., 2005). Whereas the loudness of the fundamental frequency and the third harmonic are influenced by rotational horizontal forces which vary throughout the wingbeat. Taken together, the unique aerodynamic forces of the hummingbird’s wingbeat are responsible for the relative loudness and harmonic content of the wing’s sound, giving the hummingbird’s hum its characteristic timbre (Figure 1C).

So, if we know a little bit about the forces an animal produces throughout a single wingbeat, we should be able to predict the timbre of its wing sound using this simplified model. Unlike hummingbirds, larger animals, such as parrotlets and pigeons, only produce lift forces once per wingbeat on the powerful downstroke, and not on the upstroke (Crandell and Tobalske, 2015; Hightower et al., 2017). This generates a loud wing sound that has relatively low second and fourth harmonics (Figure 1D).

Conversely, small insects like mosquitos (Figure 1E) and compact flies (Figure 1F) generate smaller aerodynamic forces but produce them on the downstroke, upstroke, and in between (Bomphrey et al., 2017). This creates more complex and louder harmonics that make the flies’ buzzing seem much louder relative to their size. In courtship displays, these tiny insects actually manipulate this relationship by shortening their strokes to generate louder, even more complex tones that attract females (Bennet-Clark and Ewing, 1968).

The model designed by Hightower et al. can be used to create detailed predictions, not only about the loudness and timbre of wing sounds, but also about the directionality of the noise and how it might be perceived over various distances. Furthermore, the model can be used to test whether wing sounds behave as would be expected. Animals that make wing sounds that do not match these predictions might be doing something unusual like using their wings for acoustic communication. Ultimately, this model will be a useful tool for studying animal behavior and the evolution of specialized wing sounds.
